# The Effectiveness of an Intensive Inpatient Psychotherapy Program for Chronic Depression: A naturalistic comparison with wait list

**DOI:** 10.1186/s12888-022-04381-5

**Published:** 2022-11-30

**Authors:** Mikkel Eielsen, Pål Gunnar Ulvenes, Jan Ivar Røssberg, Andreas Høstmælingen, Christina S. Soma, Bruce E. Wampold

**Affiliations:** 1 Institute of Clinical Medicine, University of Oslo, Sognsvanssveien 21, 0372 Oslo, Norway; 2 Research Institute, Modum Bad Psychiatric Center, Modum Bad Research Institute, Badeveien 287, 3370 Vikersund, Norway; 3 Department of Psychology, University of Oslo, Forskningsveien 3a, 0373 Oslo, Norway; 4 Department of Educational Psychology, University of Utah, 1721 Campus Center Dr., SAEC Room 3220, Salt Lake City, UT 84109 USA; 5 Department of Counseling Psychology, University of Wisconsin–Madison, 317 Education Building, 1000 Bascom Mall, Madison, WI 53706-1326 USA

**Keywords:** Intensive psychotherapy program, Psychotherapy effectiveness, Chronic depression, Inpatient treatment

## Abstract

**Background:**

Patients with chronic depression (CD) typically have an early symptom onset, more psychiatric comorbidities, more treatment attempts, and more frequent and longer inpatient hospitalizations than patients with major depressive disorders. The main purpose of this study was to investigate the effectiveness of an intensive inpatient psychotherapy program for patients with chronic depression (CD). The primary research question was whether two intensive psychodynamic inpatient treatments, affect phobia therapy (APT) and VITA, were superior to an outpatient wait list condition, receiving treatment as usual (TAU), at completion of treatment. To investigate if a potential difference between the intensive treatment and the wait list control group was dependent on a specific psychotherapeutic model, the study contrasted two therapies with similar intensity, but different theoretical rationales.

**Methods:**

Two hundred eighty patients with CD were included in a naturalistic study. Patients were assessed at four time points; assessment, start of therapy, end of therapy and 1-year follow-up. Three comparisons were performed with patients matched across groups; Intensive inpatient treatment program (APT + VITA) vs wait list during treatment, APT vs VITA during treatment and APT vs VITA during follow-up. The outcome measure was the BDI-II.

**Results:**

Intensive inpatient treatment program vs. wait list showed a significant difference in favor of the intensive treatment. No significant differences were found between APT and VITA during therapy or follow-up; but both groups had large effect sizes during treatment, which were maintained during follow-up.

**Conclusions:**

The intensive inpatient psychotherapy program showed superior effect on chronic depression over an outpatient wait list condition receiving treatment as usual (TAU), but no significant differences were found between the two intensive inpatient psychodynamic treatments. The results provide support for the effectiveness of an intensive inpatient psychotherapy program in treatment of chronic and severe disorders, such as CD, which could be of benefit for policymakers and the health care sector as they are allocating recourses efficiently.

**Trial registration:**

This study has been retrospectively registered on ClinicalTrials.gov (NCT05221567) on February 3rd, 2022.

## Background

With an estimated prevalence of 4.4% of the global population and accounting for 7.5% of all Years Lived with Disability (YLD), depressive disorders rank as the single largest contributor to non-fatal health loss [1]. To a large extent it is the recurrent nature of these disorders that accounts for the personal impact as well as the public health burden [2]. Approximately 40–50% of people experiencing a depressive episode relapse after their first episode [2, 3], and the risk for more episodes rises with each recurrence. As a consequence, some people experience a high number of episodes during their lifespan [2].

The chronicity of the disease has traditionally not only been captured by the description of the recurrence of symptoms, but also by the persistence of symptoms. In the current DSM guidelines [4] patients suffering with chronic depressive distress can either be diagnosed with persistent depressive disorder (PDD) or recurrent major depressive disorder (rMDD). Both diagnoses specify a duration of symptoms for more than 2 years. Patients diagnosed with PDD can experience low-grade chronic depression, a persistent depressive episode, or intermittent major depressive episodes. With intermittent major depressions, patients can experience up to two-month intervals of remission. On the other hand, if patients experience depressive symptoms for more than 2 years, but at the same time experience a phase of remission extending beyond 2 months, a diagnosis of rMDD is warranted [4].

This division of chronic depressed patients into two diagnostic categories based on the persistence of symptoms and the recurrence of symptoms is problematic for several reasons. First, there seems to be a lack of empirical support for differentiating between relapse of the same episode and recurrence of a new episode based on a cut off-criterion of symptom relief for more or less than 2 months [5]. Second, patients in everyday clinical practice often have a long history of illness and may struggle to remember the nature, severity and timing of their symptoms [6], which makes it difficult to verify whether previous symptom-free periods qualify as recovery or full, partial or unstable remission. Third, risk factors such as failure to seek treatment at the onset of the disorder and initial depressive- and comorbid symptom severity, predict both persistence (here defined as continuity of symptoms for at least 2 years), and recurrence of depressive episodes [7, 8]. A final, and critically important, challenge is that patients with either diagnoses typically respond poorly to treatment [9]. Accordingly, the validity of the two diagnoses has been questioned, and suggestions for combining them into one category of chronic depression (CD) has been made [10].

CD has been used to describe patients suffering from longstanding depressive distress [11, 12], thus incorporating both PDD and rMDD. Studies investigating patients with CD find that these patients typically have an early symptom onset, a more complicated treatment course, more psychiatric comorbidities, more treatment attempts, and more frequent and longer inpatient hospitalizations than patients with major depressive disorders [12]. These patients have poor functional outcomes [13], use large amounts of medical services over a long period of time, and have low rates of participation in education and work life [14–17]. Understandably these patients do not respond quickly to treatment, and thus the term treatment resistant depression (TRD) has also been used to describe their suffering and lack of benefit from treatment. However, there is no consensus on a uniform definition for TRD [18].

As different definitions for longstanding depressive disorders indicate, establishing a consensus for recognizing different forms of depressive disorders is challenging. In the current study the diagnostic group will be referred to as CD to both underline the number of episodes and/or duration of symptoms they typically experience, as well as to avoid labelling symptoms as being resistant to treatment.

A meta-analysis by Cuijpers et al. [19] found that while psychotherapy was effective for CD, the effect was probably smaller than for pharmacotherapy, whereas treatment combining psychotherapy and pharmacotherapy was more effective than either therapy alone. Meta-analyses on TRD have built on these findings; finding psychotherapy added to TAU (with pharmacotherapy) to be superior to TAU (with pharmacotherapy) [20, 21]. However, caution is warranted when interpreting TRD studies as studies including the same patient populations as CD studies, as typically the definition of TRD involves a non-response to at least one adequate trial of antidepressant treatment [20, 21]. It is not clear whether the results from trials of TRD will hold in studies with other CD populations, such as populations defined by longer durations, higher numbers of depressive episodes or treatment attempts.

Schramm et al. [9] suggested that one reason for the lack of success in treating CD, is the lack of “modular approaches to accommodate [PDD’s] multifaceted nature”. That is, treatments must be comprehensive in their approach to the various psychological and behavioral processes that contribute the disorder. While this suggestion is aimed particularly at the structure and focus of therapy, it also lends support to a separate suggestion made by Guhn et al. [22]; namely that CD patients, due to the severity of the depressive symptoms, benefit from comprehensive inpatient treatments. Guhn et al. [22] investigated the effect of cognitive behavioral analysis system of psychotherapy (CBASP) for inpatients with persistent depressive disorder (PDD) treated in a general acute psychiatric unit. The patients were offered a comprehensive inpatient psychotherapy program, with several interventions aimed at various aspects of the disorder, and the totality of the program showed notable effects in alleviating symptoms of PDD. Although there was no comparison to a control group, the study points to the potential effect of inpatient programs in treatment of chronic depressive disorders.

There is also some research indicating that dose and session frequency may be important for CD. In the meta-analysis on CD by Cuijpers et al. [19] suggested that dose of therapy was relevant for treatment outcome, and inferred that at least 18 sessions are necessary to achieve optimal treatment outcome. Session frequency may be even more important. In a meta-regresion analysis of psychotherapy for depression, Cuijpers et al. [23] found that moving from one session per week to two sessions per week improved outcomes with a moderate effect size (*d* = 0.45). Similar results were found by Bruijniks et al. [24] who compared the effects of once- versus twice-weekly sessions of cognitive-behavioral therapy (CBT) or interpersonal psychotherapy (IPT) for depression, with an increased effect of therapy by *d* = 0.55 for twice-weekly sessions.

Summarizing the findings above, the research literature suggests that psychotherapy, provided in a comprehensive inpatient program with a high frequency of interventions has promise for a CD population. This conjecture needs to be investigated in a naturalistic setting where such treatments occur, with a control condition.

This study investigated the effectiveness of an intensive (high-dose, high frequency) inpatient treatment program for patients with CD. The treatment was either affect phobia therapy (APT) [25] or the existential psychodynamic psychotherapy VITA [26] compared to treatment as usual (TAU) in the patients local communities while they were on the wait-list for the intensive inpatient treatment (i.e., wait list control). The primary research question was whether the comprehensive inpatient treatment program (APT and VITA) was superior to the wait list condition at completion of treatment. Secondary analysis tested differences between APT and VITA at termination and follow-up, to identify if outcomes of the inpatient treatment program were dependent on a specific psychotherapeutic model. The primary hypothesis was that the effect of the intensive treatment program would be superior to the wait list control group.

## Methods

### Treatment

In this study the intensive inpatient psychotherapy treatments provided were affect phobia therapy (APT) and VITA. These specific inpatient treatments have previously been described by Ulvenes et al. [27]. The patients in the wait list control group received TAU in their local communities.

### Affect phobia therapy

APT is a short-term psychotherapy (STTP) that fits within a subgroup of STTP named experiential dynamic therapy (EDT) [28]. These STTP models strongly emphasize on helping patients directly experience and express affects that have been warded-off [28]. APT views psychopathology as a consequence of the interplay between activating affects, inhibitory affects, and defenses. This interplay hampers the patients’ adaptive expression of affect and can be considered as a phobia for specific affects. Exposing the patient to warded off affects leads to desensitizing the affects, thus allowing them to be used more adaptively. APT has shown to be effective for chronic illnesses similar to CD such as cluster C personality disorders [29, 30] and has metanalytic support for psychiatric conditions in adults [28].

### Vita

The VITA model focuses on relational aspects of religious and existential issues. It’s basis is an existential and object-relational approach to psychotherapy [26] but also integrates behavioral and cognitive strategies. The treatment seeks to facilitate transformation of rigid object representations and address important existential issues such as meaning of life, shame and guilt. By resolving these existential issues, patients are expected to become less depressed, improve personality functioning, and reduce the risk of relapse. VITA has shown to be helpful to patients that qualify for a CD-diagnosis and comorbid cluster C personality disorder [31].

### Wait list control condition

The wait list control was chosen as the TAU condition for several reasons. The purpose of the study was to determine whether a high intensity comprehensive inpatient program was superior to TAU as typically provided to patients. It has been argued that TAU is the preferred control condition to estimate the effects of psychological interventions, given limitations of alternate options [32]. There is heterogeneity in TAU conditions, particularly between countries [33]. In a Norwegian context patients in TAU have access to quality mental health treatment that is available and affordable through public services, and the vast majority of patients receive outpatient treatment from a psychologist/psychiatrist and/or treatment/support from their local general practitioner. Thus, although most wait list patients in this study received treatment in a specialized mental health care environment, some patients received treatment in a primary healthcare environment. However, a recent meta-analysis by Cuijpers et al. [33] found that there were no significant differences among types of TAU for adult depression.

In this study, patients who had completed assessment and were on wait list for the comprehensive inpatient treatment program, while receiving TAU treatment locally, composed the wait list control group. In the waiting period all patients received treatment offered in their local communities, as discussed above.

In order to function as an adequate control group for the inpatient treatment, only patients who were on the wait list for the same length as the inpatient treatment (wiz. 12 weeks) were included. The mean wait list period was 28.0 weeks (*SD* = 11.7).

After these patients had finished the waiting period, serving as the wait list control, the patients were included in the intensive (high-dose, high frequency) inpatient treatment arms, receiving either APT or VITA treatment. This means that the same group of patients served as their own control group.

### General treatment information

APT and VITA psychotherapy was carried out in accordance with treatment manuals [25, 26]. In order to maintain treatment integrity, trained psychologists provided supervision for both treatments. In addition to weekly individual sessions the inpatient program at both groups contained two 75 min group sessions each week. In addition, VITA had shorter group meetings each morning (15 minutes). Patients in both treatments participated in two physical exercise sessions per week, weekly psycho-educational lectures and art-therapy groups, and both groups finish each week with end of the week status groups. On average, patients in both treatments received seven sessions of therapeutic activity each week. All treatment components, with the exception of the physical exercises, adhered to the APT or VITA treatments, and thus the two intensive treatments were similar in dose but different in content. Medication was managed by psychiatrists or medical doctors undertaking a specialization to become a psychiatrist, aiming to optimize the psychotropic medication regimen, typically by reducing antidepressant use. This is a part of the general treatment policy at the hospital. (See Table 1 for antidepressant use.)

**Table 1:** *Antidepressant use*

	Antidepressant medication			
	No		Yes	
Treatment	N	%	N	%
	Intensive Treatment vs Wait List			
Wait List	127	58.8	89	41.2
Intensive Treatment	129	59.7	87	40.3
	APT vs VITA			
APT	69	60.5	45	39.5
VITA	67	58.8	47	41.2

Note. Patients who used quetiapine and lamotrigine for antidepressant purposes have been included in the sample; Due to the matching procedure with replacement, some patients were used in multiple comparisons

### Therapists

The intensive treatment program was delivered by a treatment team. Each treatment team included at least one psychiatrist or a medical doctor specializing to become a psychiatrist, two or three psychologists and two or three nurses. In total there were 32 therapists providing the individual psychotherapy sessions, of which 62.5% were female. All therapists were Scandinavian, mainly Norwegian.

### Participants

Participants were recruited from a specialized depression treatment unit at Modum Bad, Norway. This psychiatric center is a national hospital established for the residential treatment of patients unresponsive to prior treatments. The patients included in the study have each failed treatments first by their primary care physician and then at least one attempt at their local psychiatric outpatient clinic.

The patients received treatment at the hospital between 2012 and 2017. The hospital uses an intake screening procedure whereby applications for treatment that clearly falls outside of the hospital jurisdiction is returned to the applicant or other health services. This includes patients who are not defined as needing specialized health care, suffer from disorders that the hospital has no treatment for or has not exhausted local treatment alternatives. Historical records for declined applications were not available. However, an estimation from the screening service indicates that in this period roughly 1800 patients were referred to the depression unit and about 1200 were excluded prior to assessment by the depression unit. Approximately 6 hundred patients were assessed for eligibility at the depression treatment unit, of which 437 received treatment at the unit. The patients that were excluded, did to not meet the inclusion criteria for the unit nor for this study. Thirty-seven patients were further excluded from this study due to either a bipolar diagnosis or receiving shorter treatment. Finally, a total of 120 patients were excluded from analysis because of too short waitlist period (< 12 weeks). Data from a total of 280 patients were included in the analyses.

### Inclusion criteria

To be included in the study patients had to have the characteristics of patients with chronic depression (CD), thus including patients with both PDD and rMDD. This categorization follows the rationale of studies that argue that a more valid categorization of patients with chronic depressive distress is between chronic v. non-chronic depression and patients experiencing just one or few episodes of major depressive disorder (MDD) and patients who experience a pattern of chronicity, no matter if this entails a repeated pattern of recurrent episodes or persistence of symptoms (i.e. PDD) [34]. However, a consensus on a clear cut off point between few and many episodes is yet to be established, and different studies have indicated two or more [35–37], three or more [38], or five or more [10, 39]. The patients in the current study clearly satisfies a cut-off point of two episodes or three episodes and a substantial number also qualify for a threshold of four episodes as well, as they all have exhausted local treatment alternatives including their primary care physician and the local psychiatric outpatient clinic. In addition, on average, it’s been more than 20 years since they experienced their first depressive episode. (See Table 2 for demographic and characteristics of the patients.)

**Table 2:** *Demographic and Clinical Characteristics*

	APT *N* = 133	VITA *N* = 83			Wait List *N* = 131	
Characteristic	N (SD)	%	N (SD)	%	N (SD)	%
Sex						
Women	101	75.9	60	72.3	93	71.0
Men	32	24.1	23	27.7	38	29.0
Age, years	49.6 (10.7)		46.7 (11.4)		48.4 (11.6)	
Children	93	69.9	59	71.1	86	65.6
Marital status						
Single	26	19.5	21	25.3	37	28.2
Relationship	7	5.3	2	2.4	5	3.8
Married or cohabiting	65	48.9	44	53	60	45.8
Divorced or widowed	35	26.4	16	19.3	29	22.1
Education						
Basic school (9–10 years)	14	10.5	3	3.6	12	9.2
Upper secondary school	10	7.5	9	10.8	17	13
Vocationally oriented education	18	13.5	6	7.2	14	10.7
Bachelor or higher	87	65.4	62	74.7	85	64.9
Unknown	4	3	3	3.6	3	2.3
Occupational status						
Disabled	21	15.8	6	7.2	13	9.9
Partly disabled	8	6.0	8	9.6	11	8.4
Sick leave	50	37.6	31	37.3	51	38.9
Graded sick leave	27	20.3	23	27.7	31	23.7
Fully employed	19	14.3	13	15.7	19	14.5
Student	5	3.8	2	2.4	5	3.8
Other reasons	3	2.4			1	8
Years since first episode	20.7 (14.0)		25.9 (14.3)		23.6 (13.7)	

Note. Some wait list patients were used in multiple comparisons

### Exclusion criteria

Patients were excluded if they met any of the following criteria: (a) Not having utilized reasonably available treatment in proximity to their residence, (b) having a psychotic disorder, (c) having a cluster A or B personality disorder, (d) having a bipolar disorder, (e) engaging in ongoing substance abuse, (f) having a physical brain disorder, and (g) not having access to TAU while on the 12 week wait-list period. (See Table 2 for demographic and characteristics of the patients.)

### Procedures

Patients were assessed during a 4-day evaluation stay. At the time of this assessment the majority of patients were receiving treatment from a local psychologist/psychiatrist in addition to their local general practitioner. Patients accepted for therapy returned home to receive treatment as usual during the waitlist period. After the period on the waiting list, patients were admitted to a 12-week in-patient psychotherapy program. Patients were invited to a follow-up stay at the hospital about 1-year after end of therapy. The patients provided self-reported symptoms at assessment, at end of wait list/start of therapy, termination of therapy, and at 1-year follow-up.

### Assessment

All patients were assessed and diagnosed by therapists in the two groups. This diagnosis was based on the Mini-International Neuropsychiatric Interview (M.I.N.I.) [40], Structured Clinical Interview for DSM-IV Axis II Personality Disorders (*SCID*-II) [41] as well as the self-report instrument Beck Depression Inventory-II (BDI-II) [42]. Medication, including antidepressant treatment was assessed by a psychiatrist or a medical doctor, specializing to become a psychiatrist.

### Treatment phase and 1-year follow-up

Patients were assessed with the BDI-II at start of therapy, end of therapy (12 weeks) and 1-year follow-up (1 year after end of therapy). Information regarding medication was collected at the same intervals.

### Outcomes

The primary outcome was the BDI-II, a self-report instrument for assessing severity of depression. Twenty-one items are scored on Likert scale from 0 to 4 (range 0–63). Scores between 14 and 19 indicate mild depression, 20 to 28 indicate moderate depression, and above 29 indicate major depression. The BDI-II is widely used and has demonstrated high reliability and the capacity to discriminate between depressed and non-depressed individuals, as well assessing the severity of the depression for depressed individuals [43].

### Statistics

The patients were not randomly assigned to conditions and since the study is naturalistic and not a randomized controlled trial, differences between groups could not be assumed to be randomly distributed. In order to reduce selection bias due to the lack of randomization, and to ensure comparability of the groups for baseline confounding variables, we used a matching procedure to select patients for each comparison.

### The matching procedure

In the first comparison the group of patients who received intensive psychotherapy (viz. APT and VITA) were matched with the same group of patients while they were on the wait list. In comparisons between APT and VITA patients who received APT were matched with patients receiving VITA. The matching procedure in all comparisons was propensity score matching (PSM) [44]. PSM, based on logistic regression, identifies similar patients from the groups being compared, and creates matched sets of patients. Psychometric and demographic information were used as independent variables and treatment group as dependent variable, providing a probability of treatment assignment based on observed baseline variables. The procedure matched patients according to their probability of belonging to each of the groups. That is, patients with similar scores from the logistic regression were matched if their scores fell within a predetermined range (caliber width < 0.1). In comparison 1 this approach matched each subject in the intensive treatment groups (viz. APT+VITA) to the five nearest patients in the wait list group, within the caliper width, and in comparison 2 and 3 each subject in the APT group were matched to the five nearest subjects in the VITA group within the caliper width. One patient was then selected randomly from this pool of five matches. Matching was performed with replacement, meaning that after a patient had been used as a match, the patient was not eliminated from the pool of potential new matches, meaning that each patient could be used as a match multiple times in order to create closer matching. Prior to PSM, missing values were estimated and replaced using a predictive mean matching (PMM) procedure [45] in which the missing values are first estimated using linear regressions before the estimate is replaced by the nearest observed value in the dataset. This allowed the PSM procedure to be executed on a full dataset.

### Multi-level models

Patients were assessed at four time points, yielding repeated assessments nested within patients. To account for this data structure a series of multilevel models were fitted to the data [46]. All analyses were conducted using SPSS v 25. The models were built by successively adding predictors to an empty model and tested for homo- and heteroskedastic error variance, linear- and curvilinear effect of time and piecewise effect of time. All models where tested for model fit using the − 2 log likelihood test [47]. By using full estimation maximum likelihood, models with different fixed effects were estimated. The model used for comparison 1 had fixed effects of intercept and time, and random intercept. It used scaled identity matrix as covariance structure and homoscedastic error variance. For the second and third comparison, we used a model that had fixed effects of intercept, a piecewise timeline, and random intercept. The piecewise model used two timelines; the first timeline was from start of therapy to end of therapy, and the second timeline was from end of therapy to 1-year follow-up. For comparison 2 the intercept was set at end of therapy, and for comparison 3 the intercept was set at end of follow-up. The model used scaled identity matrix as covariance structure and homoscedastic error variance.

### Comparison 1. Intensive inpatient treatment versus wait list control group (APT + VITA vs wait list control)

The sample size was *N*_APT + VITA_ = 216 (133 unique APT patients, 83 unique VITA patients), *N*_WAIT LIST_ = 216 (131 unique patients, propensity score matched with replacements). Time was centered at post-treatment (viz. end of wait list period or end of intensive inpatient treatment). The model had fixed effect of intercept, time, group (dummy coded for intensive inpatient treatment or wait list), and the interaction of time and group, and random effect of intercept.

### Comparison 2. Comparison of the two intensive inpatient treatments (APT versus VITA)

The sample size was *N*_APT_ = 114 (All unique patients) and *N*_VITA_ = 114 (83 unique patients, propensity score matched with replacements). Time was centered at post-treatment. The model had fixed effect of intercept, time, group (dummy coded for APT or VITA), and the interaction of time and group, and random effect of intercept.

### Comparison 3. APT versus VITA during follow-up

The groups were the same as the matched groups in Comparison 2. Time was centered at follow-up. The model had fixed effect of intercept, time, group (dummy coded for APT or VITA), and the interaction of time and group, and random effect of intercept.

### Effect sizes

All effect sizes (Cohen’s *d*) for the MLM-analysis were calculated based on Feingold [48].

## Results

### Comparison 1. Intensive inpatient treatment versus wait list control group (APT + VITA vs wait list control)

The between group effect size (i.e. between intensive inpatient treatment and the wait list control) was large, *d* = 0.9 in favor of the intensive inpatient treatment group.

**Fig. 1:**
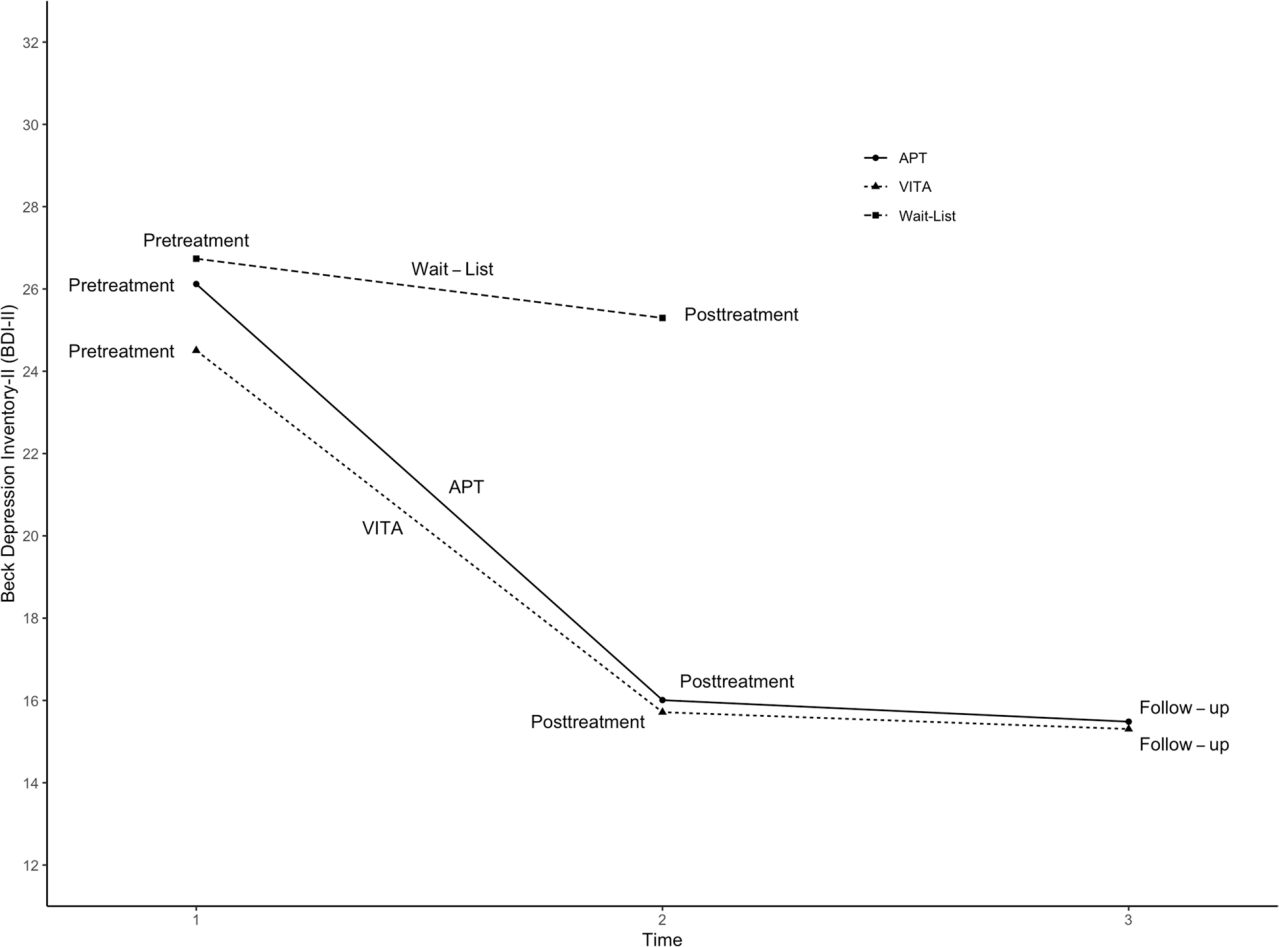
Means of Raw Scores Over the Course of Treatment for Propensity Score Matched Patients in APT, VITA and Wait List

At pre-treatment both groups scored in the upper range of moderate depression on the BDI-II (i.e., intensive inpatient treatment group: *M* = 26.2, *SD* = 9.87 and wait list: *M* = 26.7, *SD* = 9.29). However, at post-treatment the intensive inpatient treatment group scored in the lower range of mild depression on the BDI-II (*M* = 16.5, *SD* = 11.52) while the wait list patients on average still scored in the higher range of moderate depression on the BDI-II (*M* = 25.3, *SD* = 9.68). (See Fig. 1 for mean BDI-II scores in the three conditions at the various time points and Table 3 for means and standard deviations of the BDI-II for all comparisons.)

**Table 3:** *Means and Standard Deviations of the BDI-II for all Comparisons*

	Pre-treatment			Post-treatment			Follow-up		
Treatment	N	M	(SD)	N	M	(SD)	N	M	(SD)
	APT+ VITA versus Wait List								
APT+VITA	184	26.2	9.87	192	16.5	11.52			
Wait List	178	26.7	9.29	196	25.3	9.68			
	APT versus VITA								
APT	91	26.1	9.76	104	16.0	11.64	64	15.5	10.90
VITA	91	24.5	8.65	91	15.7	10.39	85	15.3	8.74

Note. Statistics were calculated based on observed data for each patient at each time point. The tests of hypotheses involve imputed data using predictive mean matching, as described in the main article

The results of the MLM analysis are displayed in Table 4. There was a statistically significant fixed effect for time, demonstrating decreasing BDI-II scores from pre-treatment to post-treatment. Further, there was a statistically significant fixed effect for group, demonstrating that the two intensive inpatient treatments were superior to the wait list condition, given that time was centered at post-treatment, and finally, a statistically significant group by time interaction, showing that the intensive inpatient treatments had a significantly steeper drop in depressive symptoms over the course of therapy compared to the control group. Not surprisingly, there was a significant random effect of intercept, indicating that the BDI-II scores at the end of treatment differed among the patients.

**Table 4:** *Results of the MLM analysis: Intensive Inpatient Treatment (APT + VITA)* vs *Wait List*

Parameter	BDI-II
	Fixed parameters
Intercept	16.1*** (.7), [14.7, 17.4]
Time	−9.9*** (.6), [−11.1, −8.7]
Group	10.7*** (.7), [9.3, 12.0]
Time * Group	8.2*** (.9) [6.5, 9,9]
	Random parameters
Intercept	71.9*** (7.9)
−2 LL	5195.2

Note. Intercept is centered at post-treatment; Standard error is given in parenthesis; 95% Confidence interval given in brackets; *p < 0.05; **p < 0.01; ***p < 0.001

Abbreviations: 2 LL, − 2 Log Likelihood; BDI-II, Beck Depression Inventory

### Comparison 2. Comparison of the two intensive inpatient treatments (APT versus VITA)

The between group effect size (i.e. between APT and VITA) was *d =* 0.0.

At pre-treatment both groups scored in the upper range of moderate depression on the BDI-II (i.e., APT (*M* = 26.1, *SD* = 9.76) and VITA (*M* = 24.5, *SD* = 8.65). Then both groups, during treatment phase, had a decline in depressive symptoms and ended up in the lower range of mild depression on the BDI-II at post-treatment (i.e., APT: *M* = 16.0, *SD* = 11.64 and VITA: *M* = 15.7, *SD* = 10.39). (See Fig. 1 for mean BDI-II scores in the three conditions at the various time points and Table 3 for means and standard deviations of the BDI-II for all comparisons.)

The results of the MLM analysis are displayed in Table 5. There was a statistically significant fixed effect for time, demonstrating decreasing BDI-II scores from pre-treatment to post-treatment, but the fixed effects for group and the interaction of group and time were nonsignificant, indicating that the two intensive inpatient treatments did not produce different effects on depression. There was a significant random effect of intercept, indicating that the BDI-II scores at the end of treatment differed among the patients.

**Table 5:** *Results of the MLM analysis: APT* vs *VITA (Treatment Phase)*

Parameter	BDI-II
	Fixed parameters
Intercept	16.0*** (1.0), [14.1, 18.0]
Time	−10.1*** (1.0), [− 12.1, − 8.1]
Group	−1.1 (1.6), [−4.3, 2.1]
Time * Group	.1 (1.5), [− 2.8, 3,0]
	Random parameters
Intercept	59.8*** (8.6)
−2 LL	3748.9

Note. Intercept is centered at post-treatment; Standard error is given in parenthesis; 95% Confidence interval given in brackets; *p < 0.05; **p < 0.01; ***p < 0.001

Abbreviations: 2 LL, − 2 Log Likelihood; BDI-II, Beck Depression Inventory

### Comparison 3. APT versus VITA during follow-up

The between group effect size (i.e. between APT and VITA) was *d* = 0.1.

As evidenced above, at post-treatment both groups scored in the lower range of mild depression on the BDI-II. During the 1-year follow-up period the patient’s depressive symptoms on average did not change much (i.e., APT: *M* = 15.5, *SD* = 10.90 and VITA: *M* = 15.3, *SD* = 8.74). (See Fig. 1 for mean BDI-II scores in the three conditions at the various time points and Table 3 for means and standard deviations of the BDI-II for all comparisons.)

The results of the MLM analysis are displayed in Table 6. The fixed effect for time was not significant, indicating that the BDI-II scores at follow-up were not different than they were at post-treatment (i.e., patients maintained the benefits of the intensive inpatient treatments). The fixed effects for group and the interaction of group and time were not significant, indicating that the two intensive inpatient treatment groups maintained benefits of treatment approximately equally. There was a significant random effect for intercept, indicating that the BDI-II scores at the end of follow-up differed among the patients.

**Table 6:** *Results of the MLM analysis: APT* vs *VITA (Follow-up)*

Parameter	BDI-II
	Fixed parameters
Intercept	16.3*** (1.2), [14.0, 18.6]
Time	.3 (1.1), [− 1.9, 2.6]
Group	−.4 (1.8), [−3.8, 3.1]
Time * Group	.7 (1.6), [− 2.4, 3,9]
	Random parameters
Intercept	59.8*** (8.6)
−2 LL	3748.9

Note. Intercept is centered at post-treatment; Standard error is given in parenthesis; 95% Confidence interval given in brackets; *p < 0.05; **p < 0.01; ***p < 0.001

Abbreviations: 2 LL, − 2 Log Likelihood; BDI-II, Beck Depression Inventory

## Discussion

The main purpose of this study was to investigate the effect of an intensive inpatient psychotherapy program for CD patients compared to a wait list control group. To investigate whether any difference between the conditions was due to a specific psychotherapeutic model, the study also contrasted two inpatient therapy programs with similar intensity, but different theoretical rationales. Thus, the study first sought out to investigate whether a CD population would benefit from a more intensive psychotherapy program. As predicted, patients with CD benefitted more from the intensive inpatient treatments than they did from quality local treatment while on the wait list. Furthermore, the analysis shows that the benefits of the intensive inpatient treatments were maintained at one-year follow-up. Both the MLM analysis and the large between group effect size support that CD can be successfully treated with intensive inpatient treatments, and these treatments are more effective than the high quality but less intensive outpatient care that is usually provided. The analysis also showed minimal differences between the two intensive inpatient treatments, suggesting that the differences in effect may not be due to the theoretical rationale within the inpatient treatment but rather the treatment context. The treatment context was in line with what was suggested by the literature as central aspects to consider in designing effective treatment for CD; comprehensive inpatient psychotherapy with a high dose and frequency.

There are several possible explanations for the observed effect. Guhn et al. [22], who investigated inpatient cognitive behavioral analysis system of psychotherapy (CBASP) for inpatients with persistent depressive disorder, suggested the large effects of the study were explained by three advantages over outpatient therapy: “(1) a high treatment frequency; (2) a combination of individual and group therapies; and (3) a high amount of corrective interpersonal experiences with different people”. Regarding high treatment frequency, Erekson et al. [49] suggested that session frequency has a direct effect on therapeutic operations (i.e. technical aspects of therapy, such as the client’s presentation of concerns, the therapist’s intervention, and the client’s cooperation with the therapist’s intervention) and the alliance between the patient and the therapist. They theorized that gains were less likely to add upon each other as time between sessions increased and found support for this in behavioral theory that suggest that continuous reinforcement is best for learning new behaviors. This could potentially be an important factor, especially in a patient group with maladaptive behaviors engrained over long time periods. Schramm et al. [9] also suggested that therapy should be modular, thus incorporating many targeted interventions equivalent to the treatment program in this study, to accommodate CD’ multifaceted nature. Similar to the treatment program in this study, Guhn et al. [22] utilized a combination of individual therapy and group therapy, which they argue makes it possible for the patients to experience a high number of corrective interpersonal experiences. In contrast to outpatient settings, the patients not only encounter corrective experiences with the therapist but also with team members and fellow patients.

There were no significant differences between the two intensive psychodynamic treatments. Thus, it seems that in this study the treatment context was more important than the psychodynamic orientation. However, both treatments in the study are psychodynamic and it cannot be ruled out that the models are too similar for any significant difference to be found. Whether other approaches offered in the same intensive format would be more effective for patients with CD is beyond the scope of this study. Also, independent of the psychotherapeutic modality, this intensive inpatient psychotherapy program includes a vast number of interventions that vary in nature, that may explain large parts of the effects. Accordingly, if the inpatient therapeutic context is a large contributor to the observed effects, identifying differences between the two treatment modalities may be less likely.

This is a naturalistic study and a strength is that the patient sample is representative of a population of patients with persistent depressive symptoms, several different treatment attempts, and lack of symptom alleviation. The results are from psychotherapy provided within standardized structured specialized mental health care. The sample included patients who exhibited symptoms and narratives of many variations of chronic depression. This increases the external validity and the applicability to a “real world practice setting.” Still, the naturalistic design of the study creates limitations, such as the lack of randomization and blinding. Also, the number of patients that were excluded prior to assessment may have contributed to a patient sample that is not representative of the CD population.

There are additional limitations to the present study. A limitation is the broad definition of Chronic Depression (CD), as well as the difficulties establishing an exact number of prior depressive episodes or the symptom duration. However, the hospital responsible for the therapy program has specialized on patients unresponsive to prior treatments. All patients assessed at the depression unit must have exhausted local treatment alternatives, including primary care physician and psychiatric outpatient clinic. This means that the hospital treats a chronic patient population, and the current patient sample reported experiencing their first symptoms more than 20 years prior to treatment. Still, both the broad limitation of CD as well as the lack of information on exact number of depressive episodes allows for unwanted heterogeneity in the patient sample.

Another limitation of the study is the lack of a control group receiving the same psychotherapy treatment, but provided with lower intensity and dose. In the current study design, one cannot rule out the possibility that a lower dose of the same treatment would have produced the same effect as the intensive treatment. However, the wait list control group in this study involved high-quality mental health services, and goes a long way to compensate for this limitation. First, the wait list patients received assessment and treatment during a short assessment stay. Second; in order for patients to be accepted into the inpatient treatment program, it was necessary for the hospital to know they were in good care with regular sessions, monitoring of deterioration, adhering to medication regimes etc., and thus the wait list group was monitored in anticipation of the inpatient treatment, and the local TAU treatment had to conform to a certain standard. Third, the patients were on the waiting list for treatment at the hospital and therefore could have experienced remoralization due to their anticipation of a much sought-after treatment [50], a phenomenon that has been linked to symptom alleviation in depression [51]. Cuijpers and colleagues [32] have made a strong case for the appropriateness of TAU as a comparison condition, albeit there appears to be heterogeneity of such conditions between countries [33]. Even though the exact nature of the treatment for each patient in the wait list condition was not monitored or assessed, given the purpose of the present study, the wait list group provided an adequate comparison condition. Although the study finds support for the notion that more sessions per week improves outcome, the naturalistic study design is not able to isolate the effect of the specific intervention components.

Another limitation of the study is that while therapists followed treatment manuals, and supervision was carried out according to these treatment manuals, systematic video evaluation of the sessions was not carried out, and therapist competency for the specific models was not assessed. This is especially problematic in comparison 2 and comparison 3, where two inpatient psychodynamic programs with similar intensity were compared. However, systematic adherence checks were not possible due to the naturalistic nature of the study, although it was expected that therapists videotaped their sessions and that these videotapes were used in supervision. Also, there was supervision of both the treatment team as a group, as well as of the individual therapist, and all supervision was provided within the theoretical rationale of the treatment modality.

## Conclusions

In this study, an intensive inpatient psychotherapy program showed superior effect on CD over a wait list control group receiving TAU. No significant differences were found between two intensive psychotherapy programs using different psychotherapeutic orientations. The study joins Guhn et al. [22] in investigating the potential impact of a comprehensive intensive inpatient therapy program for a patient group that typically suffers for extended periods of time, and where adequate symptom relief has been challenging to achieve in outpatient treatment settings. The results provide support for the effectiveness of an intensive inpatient psychotherapy program in treatment of chronic and severe disorders, such as CD, which could be of benefit for policymakers and the health care sector as they are allocating recourses efficiently.

## Data Availability

The datasets used and/or analyzed during the current study are available from the corresponding author on reasonable request.

## Abbreviations

APT: affect phobia therapy
BDI-II: Beck Depression Inventory-II
CBT: Cognitive-behavioral therapy
CD: Chronic depression
CBASP: Cognitive behavioral analysis system of psychotherapy
EDT: Experiential dynamic therapy
IPT: Interpersonal psychotherapy
MDD: Major depressive disorder
M.I.N.I.: Mini-International Neuropsychiatric Interview
PDD: Persistent depressive disorder
PMM: Predictive mean matching
PSM: Propensity score matching
rMDD: Recurrent major depressive disorder
SCID-II: Structured Clinical Interview for DSM-IV Axis II Personality Disorders
STTP: Short term psychotherapy
TAU: Treatment-as-usual
TRD: Treatment resistant depression
YLD: Years lived with disability
